# Factors influencing the development of bone starvation syndrome after total parathyroidectomy in patients with renal hyperparathyroidism

**DOI:** 10.3389/fsurg.2022.963231

**Published:** 2022-09-30

**Authors:** Xuyang Peng, Xiaofang Xia, Zhouting Li, Feng Cheng, Xi Zhu

**Affiliations:** ^1^ Department of Cardiothoracic Surgery, Lishui People’s Hospital, Lishui, China; ^2^ Department of Breast Surgery, Lishui Hospital of Zhejiang University, Lishui, China; ^3^ Department of Thyroid Surgery, Lishui Hospital of Zhejiang University, Lishui, China

**Keywords:** hyperparathyroidism, secondary, parathyroidectomy, hypocalcemia, hungry bone syndrome

## Abstract

**Purpose:**

To investigate the factors affecting the development of bone starvation syndrome (HBS) after total parathyroidectomy in patients with renal hyperparathyroidism (SHPT).

**Patients and methods:**

The clinical data and perioperative indices of 141 patients who underwent PTX for SHPT were retrospectively analyzed. The patients were divided into HBS and non-HBS groups based on postoperative minimum blood calcium <1.87 mmol/L. The differences in general clinical data and perioperative related indices between the two groups were compared; logistic regression analysis was performed to analyze the risk factors influencing HBS occurrence after surgery. Multiple linear regression method was used to analyze the factors influencing the maintenance time of intravenous calcium supplementation and total amount of calcium supplementation during intravenous calcium supplementation. The threshold value for the diagnosis of HBS was analyzed using the ROC subjects' working curve.

**Results:**

HBS occurred in 46 (32.6%) patients. Univariate analysis showed statistically significant differences in dialysis age, preoperative calcitonin, preoperative parathyroid hormone, preoperative blood phosphorus, and preoperative alkaline phosphatase between both groups (*P* < 0.05). Logistic regression analysis using stepwise entry method concluded that preoperative alkaline phosphatase was an independent factor for the development of HBS after surgery. Preoperative parathyroid hormone was an independent factor for the duration of intravenous calcium supplementation and total calcium supplementation during intravenous calcium supplementation in the HBS group. Based on the ROC curve, for postoperative HBS, the cut-off ALP value was 199.5 U/L, with a sensitivity of 80.85% and specificity of 82.61%.

**Conclusion:**

Preoperative serum ALP may be an independent factor for HBS occurrence after surgery. When preoperative ALP > 199.5 U/L, patients with SHPT are prone to HBS after surgery, and the higher the preoperative ALP, the higher the incidence of HBS, and vice versa. In addition, preoperative PTH may be the factor in the timing of postoperative intravenous calcium supplementation and the total amount of calcium supplementation during intravenous calcium supplementation in patients with HBS.

## Introduction

Parathyroidectomy (PTX) is one of the effective means of treating secondary hyperparathyroidism (SHPT) (1). Bone starvation syndrome (HBS) is a common group of clinical syndromes in patients with SHPT, in the context of skeletal hyper conversion state, after PTX, with severe hypocalcemia (SH) as the main manifestation and its incidence ranging from 27.4% to 87.8% (2, 3), with life-threatening arrhythmias, cardiac arrest, congestive heart failure, and laryngeal and respiratory muscle spasms in severe cases (4). The state of bone starvation after PTX is a marker of both success of the procedure and chance that the patient's skeletal lesions can be repaired. Currently, HBS mostly relies on empirical protocols for maintaining electrolyte balance, lacking sufficient attention, awareness, and individualized treatment strategies.

In this study, we retrospectively analyzed the clinical data of all patients with SHPT who underwent successful PTX in our center to study the factors influencing perioperative bone starvation syndrome and to empirically determine the timing and dosage of postoperative calcium supplementation to provide a theoretical basis for identifying and preventing HBS and reducing HBS occurrence in perioperative patients.

## Material and methods

### General data

The clinical data of 141 patients who underwent total PTX with forearm auto transplantation (tPTX + AT) for renal hyperparathyroidism at Lishui Hospital, Zhejiang University from August 2015 to November 2021 were retrospectively analyzed. Among them, 68 were male and 73 were female, with average age of 49.81 ± 7.83 years. All patients received maintenance dialysis treatment; 61 and 80 cases received peritoneal dialysis and hemodialysis, respectively. The study was approved by the Ethics Committee of Lishui Central Hospital, Zhejiang Province (no. 2022-06). All patients provided written informed consent and were included in the study (Table 1).

**Table 1:** Comparison of preoperative and postoperative demographic and laboratory data in maintenance dialysis patients with PTX + AT.

Variable	HBS group (*n* = 46)	Non-HBS group (*n* = 95)	*P* value
Gender (M/F)	29/17	39/56	<0.05*
Age (years)	47.57 ± 7.73	50.89 ± 8.02	0.060
Height (meter)	161.85 ± 5.77	160.17 ± 6.14	0.208
Weight (kg)	58.99 ± 8.66	56.11 ± 7.85	0.118
Systolic pressure (mmHg)	147.76 ± 19.76	142.67 ± 19.89	0.259
Diastolic pressure (mmHg)	87.93 ± 11.28	86.16 ± 11.05	0.488
Preoperative laboratory parameters			
Hb (g/L)	104.57 ± 14.43	106.14 ± 13.11	0.630
HCT	0.31 ± 0.05	0.32 ± 0.04	0.247
GLU (mmol/L)	4.70 ± 0.73	5.11 ± 1.19	0.575
TG (mmol/L)	1.85 ± 0.90	1.83 ± 0.82	0.790
TC (mmol/L)	4.23 ± 0.84	4.53 ± 0.88	0.125
HDL (mmol/L)	0.98 ± 0.25	1.02 ± 0.22	0.404
LDL (mmol/L)	2.13 ± 0.51	2.27 ± 0.56	0.270
iPTH (pg/ml)	1878.39 ± 665.70	1432.63 ± 391.22	<0.05*
CT (pg/ml)	14.78 ± 14.02	7.04 ± 5.69	<0.05*
Albumin (g/L)	36.15 ± 2.79	36.35 ± 2.83	0.753
ALP (U/L)	444.48 ± 249.28	153.69 ± 54.91	<0.05*
Ca (mmol/L)	2.35 ± 0.13	2.41 ± 0.15	0.069
*P* (mmol/L)	2.29 ± 0.45	2.06 ± 0.43	<0.05*
K (mmol/L)	4.45 ± 0.73	4.43 ± 0.83	0.842
Postoperative laboratory parameters			
iPTH (pg/ml)	37.72 ± 23.92	46.09 ± 42.22	0.339
Ca (mmol/L) (Day 1)	1.75 ± 0.15	2.21 ± 0.18	<0.05*
Ca (mmol/L) (Day 3)	1.68 ± 0.16	2.34 ± 0.24	<0.05*
*P* (mmol/L) (Day 1)	1.64 ± 0.36	1.67 ± 0.35	0.788
*P* (mmol/L) (Day 3)	1.34 ± 0.33	1.37 ± 0.30	0.661
ALP (U/L) (Day 3)	20.83 ± 15.12	15.98 ± 12.11	0.054
Postoperative situation Duration of intravenous calcium therapy	4 (3, 5)	1 (1, 2)	<0.001*
Postoperative hospital stay	4 (4, 5)	4 (4, 5)	0.020*

HBS, hungry bone syndrome; non-HBS, non-hungry bone syndrome; Hb, hemoglobin; HCT, hematocrit; GLU, blood glucose; TG, triglyceride; TC, total cholesterol; HDL, high-density lipoprotein; LDL, low-density lipoprotein; Ca, serum calcium; P, serum phosphorus; iPTH, intact parathyroid hormone; ALP, alkaline phosphatase.

* *P* < 0.05.

### Indications and contraindications for surgery

The indications for PTX surgery were based on the KDIGO 2017 Clinical Practice Guideline Update for the Diagnosis, Evaluation, Prevention, and Treatment of Chronic Kidney Disease–Mineral and Bone Disorder (CKD-MBD) (1). One of the following conditions had to be satisfied for PTX: (1) persistent blood whole segment parathyroid hormone (iPTH) >800 ng/L on maintenance dialysis; (2) persistent hypercalcemia and/or hyperphosphatemia refractory to drug therapy; and (3) imaging evidence of at least one enlarged parathyroid gland.

Inclusion criteria for this study were (1) age ≥18 years, on maintenance dialysis for >3 months; (2) successful tPTX + AT with ≥3 glands resected and peripheral blood iPTH <60 ng/L, 24 h after surgery (5).

**Figure 1:**
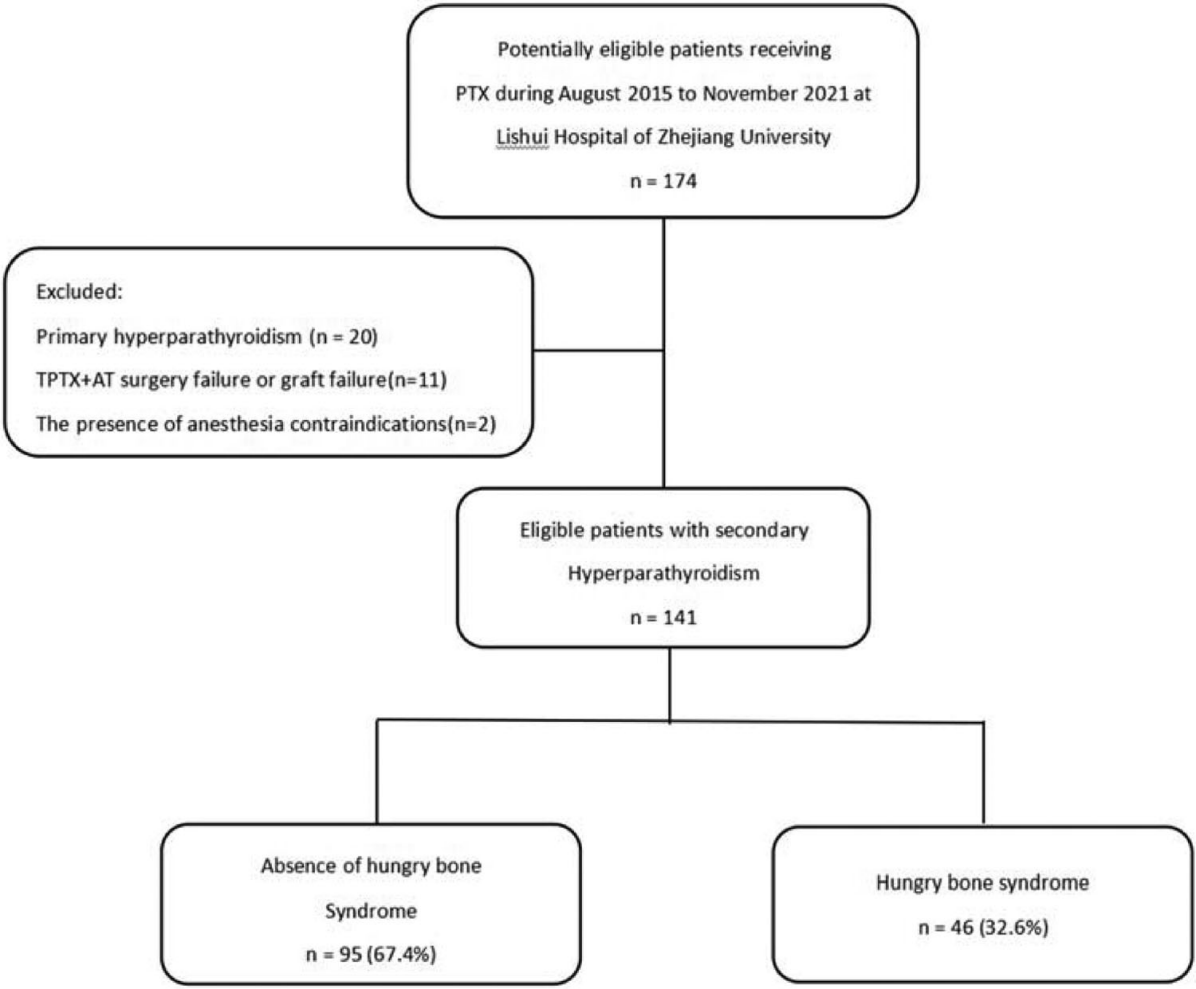
Subject disposition for the study cohort.

Exclusion criteria included (1) primary hyperparathyroidism; (2) failed tPTX + AT or non-viable grafts; (3) the presence of contraindications to anesthesia (Figure 1).

### Surgical procedure

All patients in the group were adequately dialyzed before surgery, and patients undergoing hemodialysis were dialyzed three times a week for 4 h each time with regular hemodialysis. Heparin-free dialysis was performed a day before surgery and a week after surgery. Peritoneal dialysis was performed daily, and the dialysis fluid was changed four times a day with 2000ml each time and once every hour. The dialysis fluid was kept in the abdominal cavity at night. All patients underwent tPTX + AT under sedation-combination anesthesia after completion of routine preoperative examination to exclude contraindications to surgery (the procedures were performed by trained physicians from the same treatment group). The postoperative calcium supplementation protocol: (1) non-HBS group: The initial calcium supplementation dose was 9 g of calcium carbonate tablets and 1.5 ug of calcitriol three times a day orally, and 10% calcium gluconate solution was given three times a day intravenously at 20 ml/h. (2) HBS group: On the basis of calcium supplementation in the non-SH group, the frequency and rate of intravenous calcium supplementation were adjusted to maintain the blood calcium above 1.87 mmol/L, when the blood calcium level was lower than 1.87 mmol/L in postoperative monitoring. No symptoms of hypocalcemia, such as numbness and tingling around the mouth or fingertips, were present.

The demographic variables (including age, sex, dialysis mode, dialysis age, blood pressure, height and weight), preoperative laboratory indices (including hemoglobin, fasting glucose, triglycerides, total cholesterol, serum albumin, alkaline phosphatase, blood potassium, blood calcium, blood phosphorus, parathyroid hormone, and calcitonin), and postoperative laboratory indices (including postoperative blood calcium, blood phosphorus, blood potassium, and alkaline phosphatase) of all enrolled patients were recorded. Additionally, postoperative intravenous calcium maintenance time, postoperative intravenous calcium level, and oral calcium level during intravenous calcium supplementation were noted.

Early severe hypocalcemia was defined as free calcium ≤0.8 mmol/L (≤3.2 mg/dl) and albumin-corrected total calcium ≤1.87 mmol/L (7.5 mg/dl) (6). All blood calcium level values were expressed using corrected calcium.

Total calcium supplementation during intravenous calcium supplementation = intravenous calcium supplementation during hospitalization + oral calcium supplementation during intravenous calcium supplementation.

### Statistical analysis

All statistical analyses were performed using SPSS 21.0 software. Normally distributed measurement data are expressed as mean ± standard deviation, and evaluated using independent samples *t*-test. Whereas non-normally distributed measurement data are expressed as median (25% quantile, 75% quantile), and evaluated using Mann-Whitney *U* test. Count data are expressed as frequencies (%), and evaluated using the chi-square test. Correlations between normal continuous variables were analyzed using Pearson's correlation analysis, otherwise Spearman correlation analysis was used. Logistic regression analysis and multiple linear regression analysis were used to analyze the factors associated with postoperative HBS. Subject work characteristic (ROC) curve analysis was used to evaluate the diagnostic value of the selected variables. Differences were considered statistically significant at a *P*-value <0.05.

## Results

**Figure 2:**
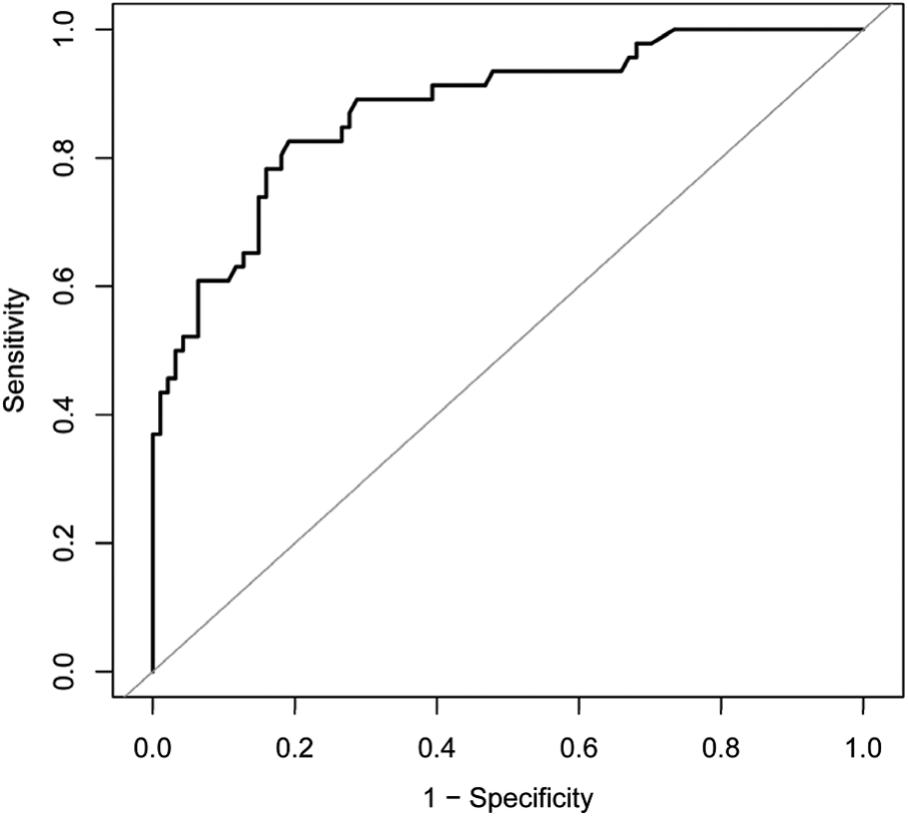
ROC curve for preoperative serum ALP receiver operating characteristic; ALP, alkaline phosphatase; AUC, area under the curve.

As reported in (Table 1), 141 patients were included in this study; 46 patients in the HBS group accounted for 32.6% of all patients. The differences in dialysis age, preoperative calcitonin, preoperative parathyroid hormone, preoperative blood phosphorus, and preoperative alkaline phosphatase between the HBS and non-HBS groups were statistically significant (*P* < 0.05). Specifically, the HBS group had higher dialysis age, preoperative calcitonin, preoperative parathyroid hormone, preoperative blood phosphorus, and preoperative alkaline phosphatase than the non-HBS group. Additionally, dialysis age, preoperative calcitonin, preoperative parathyroid hormone, preoperative blood phosphorus, and preoperative alkaline phosphatase were significantly higher in the HBS group than in the non-HBS group. Dialysis age, preoperative calcitonin, preoperative parathyroid hormone, preoperative blood phosphorus, and preoperative alkaline phosphatase were considered as independent variables, and postoperative HBS was considered as the dependent variable. Logistic regression analysis was performed using the stepwise entry method, and the results are shown in (Table 2). A higher preoperative alkaline phosphatase resulted in a higher likelihood of postoperative HBS. Pearson or Spearman correlation analysis was used to analyze the correlation among intravenous calcium supplementation time, total intravenous calcium supplementation, and various factors in the HBS grouphe results suggested that a correlation existed between intravenous calcium supplementation time and dialysis age (*r* = 0.345, *P* = 0.022), preoperative parathyroid hormone (*r* = 0.325, *P* = 0.029), and preoperative alkaline phosphatase (*r* = 0.343, *P* = 0.020).The total intravenous calcium supplementation in the HBS group had a correlation with preoperative parathyroid hormone (*r* = 0.405, *P* = 0.006) and preoperative alkaline phosphatase (*r* = 0.291, *P* = 0.049). The multiple linear stepwise regression analysis included time of intravenous calcium supplementation and total calcium supplementation during intravenous calcium supplementation in the HBS group as dependent variables and the above correlated indicators as independent variables. The analysis revealed that preoperative parathyroid hormone was an independent factor influencing the time of intravenous calcium supplementation and total calcium supplementation during intravenous calcium supplementation in the HBS group. A higher preoperative parathyroid hormone level resulted in a longer time of intravenous calcium supplementation and higher total intravenous calcium supplementation level in patients with HBS, as shown in (Table 3). There were significant differences in the duration of intravenous calcium treatment and postoperative hospital stay between the HBS group and the non-HBS group (*P* < 0.05). The duration of intravenous calcium treatment and postoperative hospital stay in the HBS group were significantly higher than those in the non-HBS group (Table 1).Finally, the ROC curve was used to evaluate the potential application of plasma metabolite profile as a biomarker for preoperative diagnosis of postoperative HBS. Based on the ROC curve, the threshold value of ALP was 199.5 U/L. The sensitivity of preoperative serum ALP was 80.85%, the specificity was 82.61%, the AUC was 0.871, and the threshold value of ALP was 199.5 U/L (Figure 2).

**Table 2:** Influencing factors of postoperative HBS (logistic regression analysis).

Variable	OR	95% CI	*P* value
Constant	−5.10	0.006	<0.01
PreALP (IU/L)	0.012	1.012 (1.006–1.017)	<0.01*

ALP, alkaline phosphatase; OR, odds ratio; CI, confidence interval.

* *P* < 0.05.

**Table 3:** Influence factors of maintenance time and total amount of intravenous calcium supplementation in patients with hypocalcemia (multiple linear stepwise regression analysis).

Dependent variable	Independent variable	Beta	*P* value
Time of intravenous calcium supplementation (days) (*F* = 5.010, *R*^2^ = 0.109, *P* = 0.031)	Constant	2.533	0.001
	Pre-iPTH	0.001	0.031*
Total amount of calcium during intravenous calcium supplementation (mmol/L) (*F* = 11.453, *R*^2^ = 0.210, *P* = 0.002)	Constant	545.756	0.010
	Pre-iPTH	0.340	0.002*

iPTH, intact parathyroid hormone.

* *P* < 0.05.

## Discussion

SHPT is one of the common complications of stage 5 chronic kidney disease (CKD-5) (7). Severe SHPT is associated with skeletal deformities, bone pain, muscle weakness, pruritus, atrophy, restless leg syndrome, dry syndrome, ectopic calcification, constipation, peptic ulcer disease, spontaneous long fractures, and sleep disturbances, which severely affect the quality of life of patients. According to reports, about 15% of patients need PTX after 10 years of dialysis and 38% of patients need surgery after 20 years of dialysis (8). Surgery can reduce iPTH, correct electrolyte disturbances, and improve prognosis.

iPTH is established as one of the most important regulatory hormones in the process of skeletal reconstruction, and physiological doses of iPTH increase bone formation and are used as targets in designing osteoporosis treatment drugs (9). However, when iPTH exceeds the physiological requirement, the abnormally elevated iPTH indirectly increases the number and activity of osteoclasts through its effect on osteoblasts and osteoclasts (10). Bone resorption by osteoclasts is enhanced, and excess calcium, phosphorus, other minerals, and type I collagen breakdown products are released into blood. These changes manifest as hypercalcemia, hypophosphatemia, and elevated serum bone conversion indices, if they cannot be excreted through the kidneys in a timely manner. Concurrently, the activity of osteoblasts is enhanced. However, due to the enhanced activity of osteoclasts in the hyperparathyroid phase and abnormal calcium and phosphorus factors, sufficient hydroxyapatite cannot be formed or effectively deposited in the damaged bone. As a result, the number of bones failing to mineralize effectively increases in most patients with SHPT. After PTX, iPTH decreases sharply, osteoclast function decreases abruptly, osteoblast activity increases, and blood calcium is absorbed into the bone (11). Electrolyte balance is severely disturbed, resulting in the “bone starvation syndrome”. Therefore, the basic principle of HBS treatment is to provide sufficient raw materials for bone repair to achieve the optimal state of bone recovery, while maintaining stable blood calcium and phosphorus indices to minimize postoperative complications and shorten hospital stay.

Our study showed that HBS occurred in 32.6% (46/141) of patients with SHPT after tPTX + AT, which is basically similar to 27.4%–87.8% in previous literature (2, 3). Additionally, preoperative ALP elevation was found to be a risk factor for postoperative HBS. Gong et al. (12), Tsai et al. (13) and Gao et al. (14) showed in literature that age, preoperative calcium, ALP, and iPTH concentration are associated with HBS. Gong et al. (12) reported that advanced age is a risk factor for postoperative HBS, but Jakubauskas et al. (15) concluded that the younger the age, the higher the risk of HBS. However, age and preoperative blood calcium were not demonstrated to be different between the two groups in this study. Furthermore, although our initial univariate analysis showed a significant difference in iPTH in the HBS group, when adjusted in a multivariate model, iPTH was not found to be a significant independent predictor. The importance of iPTH in patients with SHPT cannot be denied, but the inclusion of patients who were treated preoperatively with concomitant calcium-mimetic and phosphorus-binding agents and other related medications that can reduce iPTH, which may have an impact on the final results, compared with previous studies, is consistent with the relative complexity encountered in our clinical work. In addition, the threshold value of ALP was calculated to predict HBS. A linear curve was used to analyze the correlation, which showed a negative correlation (*r* = 0.3389). The ROC curve indicated that the threshold ALP value was 199.5 U/L, and when preoperative ALP > 199.5 U/L, the incidence of postoperative HBS increases significantly, and vice versa, the incidence decreases. This finding can provide clinicians with a basis for early identification of patients with postoperative HBS.

Analysis of the timing and amount of calcium supplementation in patients with HBS showed that preoperative iPTH was an independent factor affecting the timing of postoperative intravenous calcium supplementation and total calcium supplementation during intravenous calcium supplementation in patients with HBS, with a positive correlation between the two factors. Although few studies on this subject have been reported, one study (16) showed that, regardless of the type of hyperparathyroidism, bone resorption indices decreased significantly, whereas bone formation markers, such as ALP, remained unchanged or even increased in patients with HBS at 72 h after PTX. Kang et al. (17) reported 91 patients with SHPT and PTX, and PTX surgery was not single, including total resection, subtotal resection and partial resection. The results showed that preoperative blood iPTH and ALP and the decrease of blood phosphorus 48 h after surgery were independent influencing factors of total calcium supplementation during postoperative hospitalization. Wong et al. (18) and Goh et al. (19) showed that postoperative elemental calcium requirements were positively correlated with preoperative ALP levels. Additionally, Wong et al. (18) specified a detailed calcium supplementation regimen based on ALP levels, which reduced the risk of postoperative hypocalcemia and shortened the length of hospital stay, while increasing the rate of transient hypercalcemia to some extent. Contrary to previous studies, although this study demonstrated a correlation among dialysis age, preoperative parathyroid hormone, preoperative alkaline phosphatase, and postoperative calcium supplementation, further multiple linear stepwise regression analyses showed that iPTH had an independent effect on postoperative intravenous calcium supplementation time and total intravenous calcium supplementation during intravenous calcium supplementation, with higher preoperative parathyroid hormone levels in patients with HBS having longer intravenous calcium supplementation time and higher total intravenous calcium supplementation. The rapid decrease in ALP suggests that ALP-mediated bone formation reached relative stability within a short period. Concurrently, the abrupt decrease in iPTH resulted in significantly decreased osteoclast number and activity, diminished bone resorption, increased osteoblast responsiveness, and a sustained decrease in blood calcium (11). As observed in *in vitro* experiments performed by Liu et al. (20), when osteoblast cell lines were cultured at consistently high PTH concentrations followed by a sudden withdrawal of PTH, the calcium and phosphorus content of the culture fluid decreased rapidly and mineralized nodules increased. Therefore, preoperative iPTH levels are more significant than preoperative ALP for the total duration of postoperative calcium supplementation and total amount of calcium supplementation.

The study had few limitations. First, due to the prophylactic nature of our postoperative protocol, the natural progression of HBS in the absence of calcium supplementation could not be observed, which may have had an inhibitory effect on the results. Second, HBS is a syndrome with a complex clinical presentation that should include the presence of low blood phosphorus, low blood magnesium, and evidence of skeletal involvement (including clinical signs, symptoms, bone mineral density, and bone resorption) in addition to the patient's electrolyte disturbances, such as blood calcium levels. These factors will be considered in further studies conducted subsequently.

## Data Availability

The original contributions presented in the study are included in the article/Supplementary Material, further inquiries can be directed to the corresponding author/s.
