# Supplementary material for: Factors influencing the development of bone starvation syndrome after total parathyroidectomy in patients with renal hyperparathyroidism
Source: Front Surg. 2022 Sep 30;9:963231. doi: 10.3389/fsurg.2022.963231 (PMC9563016; doi:10.3389/fsurg.2022.963231)
Supplement: Supplementary file 1 [file DataSheet1.docx]

Supplementary Material

# Supplementary Figures
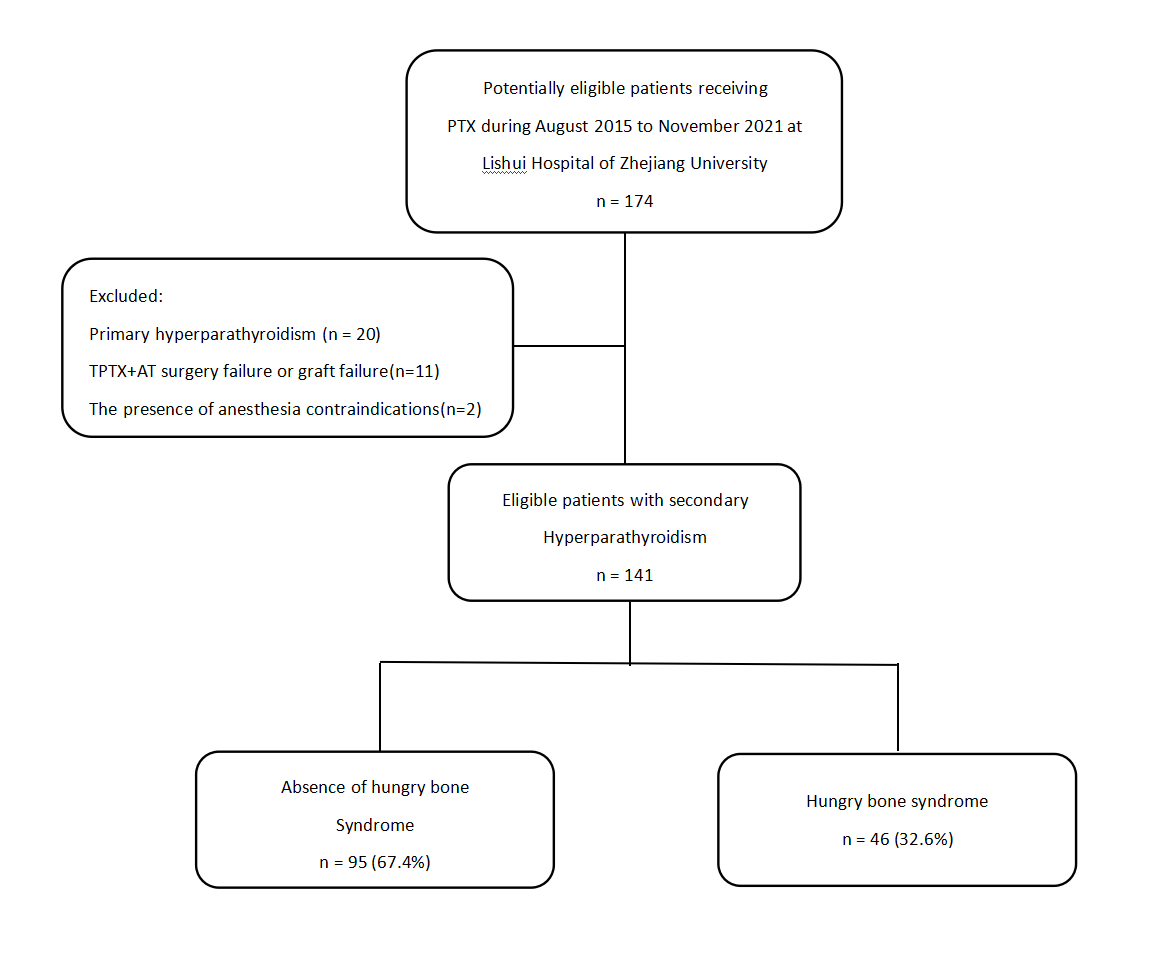


**Supplementary Figure 1.** Subject disposition for the study cohort.


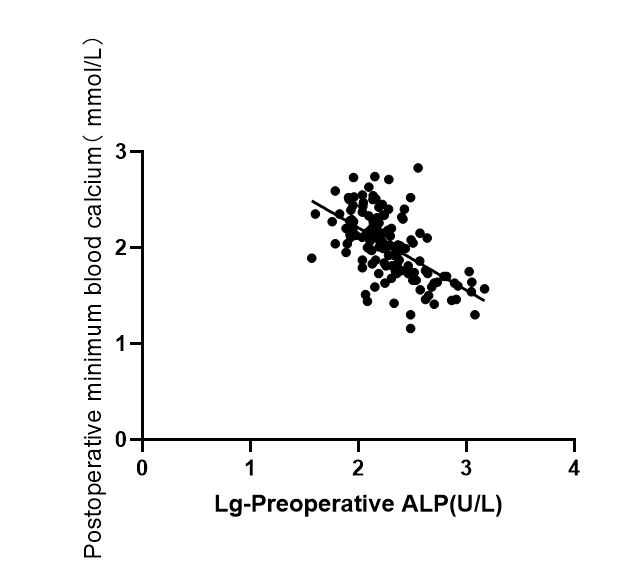


**Supplementary Figure 2.** Linear regression between Lg-Preoperative ALP(a) and postoperative minimum serum calcium.  Linear regression analysis showed that LG-ALP was negatively correlated with postoperative calcium content, with correlation coefficients of 0.3389 (P < 0.0001).

**
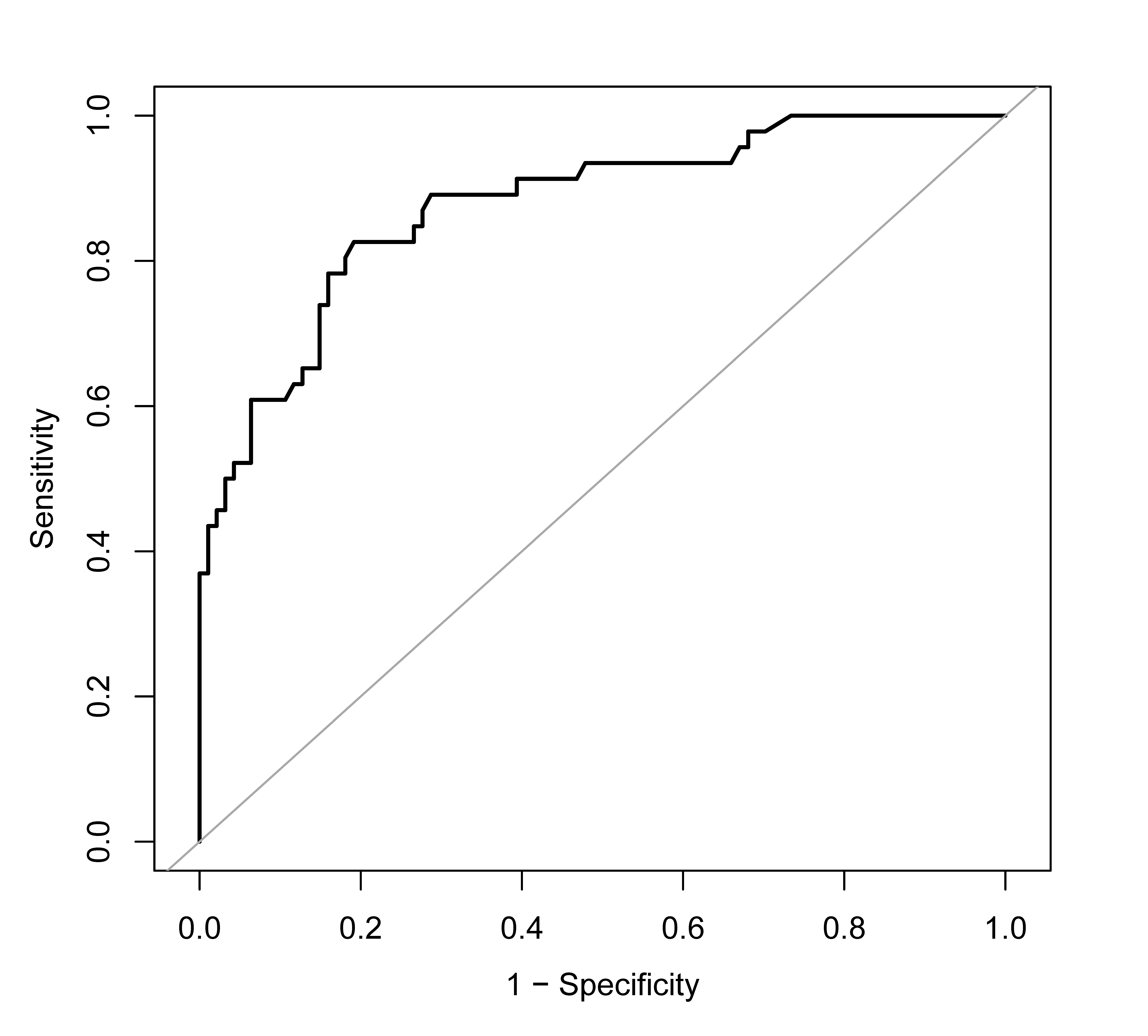
**

**Supplementary Figure 3.** ROC curve for preoperative serum ALP receiver operating characteristic; ALP, alkaline phosphatase; AUC, area under the curve.
